# Roles of LncRNAs in Viral Infections

**DOI:** 10.3389/fcimb.2017.00205

**Published:** 2017-05-26

**Authors:** Weiwei Liu, Chan Ding

**Affiliations:** Avian infectious Department, Shanghai Veterinary Research Institute, Chinese Academy of Agricultural ScienceShanghai, China

**Keywords:** long non-coding RNAs, virus infection, cellular lncRNAs, virus-encoded lncRNAs, cell-virus interaction

## Abstract

Many proteins and signaling pathways participate in anti-viral host responses. Long non-coding RNAs (lncRNAs), a subset of non-coding RNAs greater than 200 nucleotides in length, have been recently described as critical regulators in viral infections. Accumulating research indicates that lncRNAs are important in the development and progression of infectious diseases. LncRNAs are not only involved in anti-viral responses, but in many different virus-host interactions, some of which may be beneficial to the virus. Here we review the current knowledge regarding host and viral lncRNAs and their roles in viral infections. In addition, the potential of using lncRNAs as diagnostic biomarkers is discussed.

## Introduction

Fewer than 2% of genes are transcribed into mRNAs. A large number of non-coding RNAs (ncRNAs) also play important cellular functions. Based on their length, ncRNAs can be broadly classified as either short ncRNAs (<200 nucleotides) or long ncRNAs (>200 nucleotides, i.e., lncRNAs). Short ncRNAs can be further classified as small interfering RNAs (siRNAs), microRNAs (miRNAs), and Piwi-interacting RNAs (piRNAs). MiRNAs are the best characterized ncRNAs and are well known to induce mRNA degradation or inhibit mRNA translation *via* the RNA interference pathway. Compared with miRNA, much less is known about the function of lncRNAs.

LncRNAs are the transcribed and spliced products of RNA polymerase II or III transcription, are 5′capped, and may contain a polyadenylated tail at the 3′end. The expression of lncRNAs is much lower in comparison to mRNAs and lncRNAs are expressed in cell-, tissue-, and developmental stage-specific manners (Djebali et al., 2012). According to their position relative to the neighboring protein-coding gene, lncRNAs are classified as sense, antisense, bidirectional, intronic, or intergenic. The human genome encodes thousands of lncRNAs. Previously, lncRNAs were considered as “dark matter” or “junk” in the genome (Doolittle, 2013). However, recent studies have illuminated the roles of lncRNAs, and they are now considered important physiological regulators of cell homeostasis, growth, and differentiation (Wapinski and Chang, 2011; Hu et al., 2012; Fatica and Bozzoni, 2014). Emerging data have also identified the important roles of lncRNAs in regulating anti-viral responses. This review highlights specific lncRNAs associated with viral infection, specifically focusing on their expression and function.

## Functions and mechanisms

LncRNAs regulate numerous cellular processes such as gene imprinting, regulation of the p53 pathway, stem cell self-renewal and differentiation, and DNA damage response (Latos et al., 2012; Liu et al., 2013; Yang et al., 2014; Sharma et al., 2015). MiRNAs (about 19–25 nt in length) are known to take part in many of these cellular activities (Ameres and Zamore, 2013; Ha and Kim, 2014). MiRNAs modulate mRNA degradation or translation by base-pairing to sequence motifs of mRNAs. In contrast, lncRNAs utilize a multitude of mechanisms, mediated by their specific sequences or structural motifs that bind DNA, RNA, or protein. LncRNAs can function *in cis* to regulate expression of a neighboring gene and *in trans* to impact gene expression across chromosomes. Furthermore, lncRNAs function as signals, decoys, guides, and scaffolds to regulate different processes, ranging from chromatin remodeling, transcription, to post-transcriptional regulation (Wang and Chang, 2011; Bonasio and Shiekhattar, 2014).

### Chromatin remodeling

**Figure 1:**
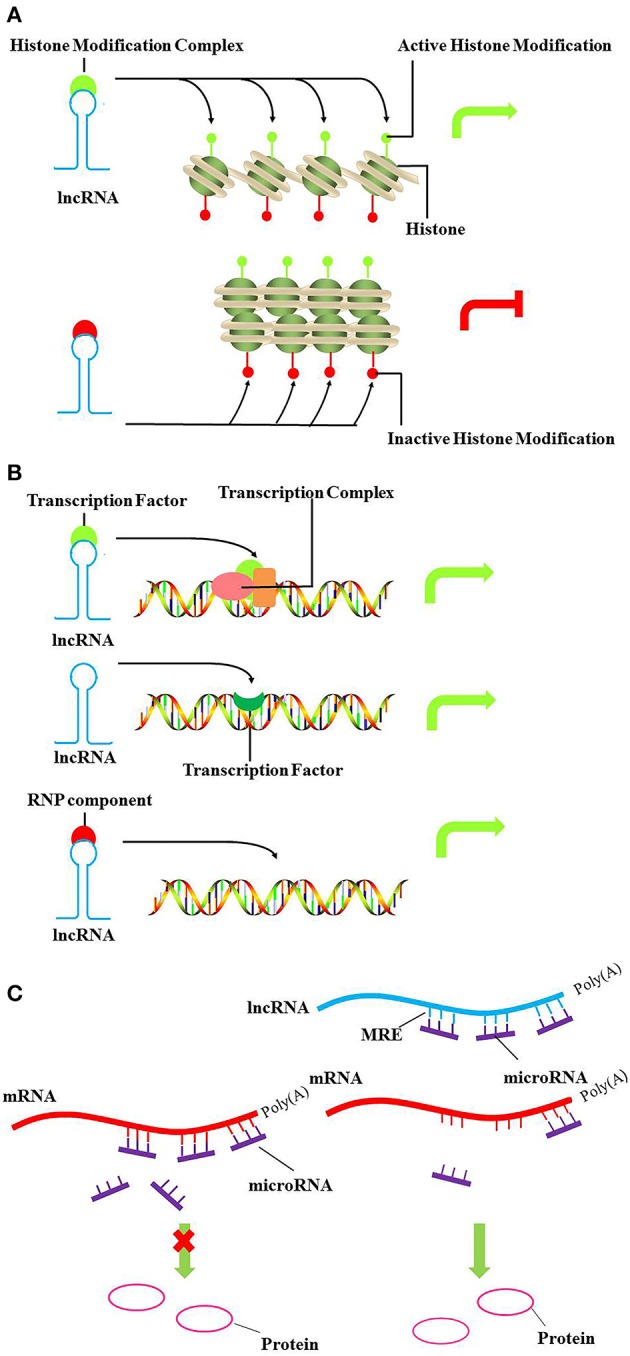
**Models of lncRNA mediated chromatin remodeling, transcriptional, and post-transcriptional regulation. (A)** LncRNA can act as scaffold to recruit chromatin remodeling components, such as histone modifiers to specific genomic loci and reprogram the state of chromatin to silence or activate transcription. The upper panel and lower panel represent the active and inactive chromatin, respectively. **(B)** In the upper panel and lower panel, lncRNA can act as decoy or as a guide to bind transcription factors or ribonucleoproteins, altering recruitment to specific genomic loci *in cis* or *in trans*, ultimately driving transcription of the localized gene. As shown in the middle panel, lncRNA can also act as signal to regulate gene expression. **(C)** LncRNAs act as miRNA “sponges” by sharing common MREs, inhibiting normal miRNA targeting activity on mRNA. Green arrows, activate transcription; Red arrows, inhibit transcription.

DNA methylation and histone modifications can alter the state of chromatin, resulting in transcriptional activation or silencing. In this setting, lncRNA recruits chromatin remodeling components to specific genomic loci, reprogramming the state of chromatin to silence or activate transcription (Figure 1A). For example, the Hox transcript antisense intergenic RNA (HOTAIR) is an lncRNA expressed from the developmental HOXC locus that can serve as a scaffold to recruit PRC2 and LSD1 *in trans*, leading to H3K27 methylation and H3K4me2 demethylation. (Gupta et al., 2010; Tsai et al., 2010). H3K27 methylation is associated with transcription repression, while H3K4me2 demethylation is associated with transcription activation. Furthermore, lncRNAs can also regulate expression of neighboring genes *in cis*, especially in imprinting. The lncRNA Air is imprinted and expressed only from the paternal allele, which at the promoter of Slc22a3 recruits G9a and leads to targeted H3K9 methylation and allelic silencing (Nagano et al., 2008).

### Transcriptional regulation

LncRNAs also act as decoys, signals or guides to play important roles in transcriptional regulation (Figure 1B). Recently, the lncRNA, Lethe, was identified as a pseudogene. Lethe is upregulated directly by NF-κB after stimulation with TNF-α or glucocorticoid receptor dexamethasone. Furthermore, as a decoy, Lethe can bind to RelA–RelA homodimers and block binding to other NF-κB response elements, thus inhibiting the function of NF-κB, and leading to decreased expression of downstream effectors, such as IL-6, SOD2, IL-8, and NF-κB (Rapicavoli et al., 2013). Moreover, lncRNA THRIL, and lncRNA-Cox2 regulate the transcription of TNF-α and CCL5 by binding hnRNP (heterogeneous nuclear ribonucleoprotein) isoforms (Carpenter et al., 2013; Li et al., 2014).

### Post-transcriptional regulation

LncRNAs also participate in post-transcriptional regulatory networks. LncRNAs have recently been suggested to act as miRNA “sponges” by sharing common miRNA response elements (MREs) and inhibiting normal miRNA targeting activities on mRNA (Figure 1C). Competing endogenous RNAs (ceRNAs) vie with mRNAs for miRNAs with shared MREs and act as modulators of miRNA by influencing the available amount of miRNA(Sen et al., 2014). Linc-MD1 is a cytoplasmic lncRNA expressed during myoblast differentiation that acts as a ceRNA for miR-133 and miR-135 to control MEF2C, MAML1 and myoblast differentiation (Cesana et al., 2011).

## LncRNA and virus infection

Recently, lncRNAs have been shown to exert both positive and negative effects on innate immunity and virus replication (Ding et al., 2016; Fortes and Morris, 2016). Next, we will discuss the cellular lncRNAs in virus-infected cells, virus-encoded lncRNAs and chimeric lncRNAs formed by viral and cellular sequences, respectively (Table 1).

**Table 1:** **LncRNAs in virus infection**.

**lncRNA**	**Virus**	**Differential expression**	**Neighboring coding genes**	**Sub location**	**Characteristics/functions**	**References**
**CELLULAR LncRNAS IN VIRUS-INFECTED CELLS**						
lncRNA-CMPK2	HCV	Up	CMPK2	Nucleus	Negative regulator of IFN responses; promotes virus replication	Kambara et al., 2014
NRAV	IAV	Down	dynll1	Nucleus	Promotes influenza A virus (IAV) replication and virulence; negatively regulates the initial transcription of multiple critical IFN-stimulated genes (ISGs), including IFITM3 and MxA, by affecting histone modifications	Ouyang et al., 2014
NeST	Theiler's virus	Up	IFN-γ	Nucleus	Increases Theiler's virus persistence and decreases *Salmonella enterica* pathogenesis. Binds WDR5 to alter histone 3 methylation at the IFN-γ locus	Bihl et al., 1999; Gomez et al., 2013
NRON	HIV	Down	MVB12B	Cytoplasm	Binds transcriptional regulators as a scaffold	Willingham et al., 2005; Sharma et al., 2011; Imam et al., 2015
NEAT1	Japanese encephalitis and rabies virus, HIV, influenza virus and herpes simplex virus	Up	FRMD8, MIR612	Nucleus	Serves as a structural scaffold for the formation of nuclear paraspeckles; enhances HIV production; facilitates the expression of antiviral genes including cytokines such as IL-8 by cooperative action of NEAT1 and SFPQ	Guru et al., 1997; Saha et al., 2006; Bond and Fox, 2009; Clemson et al., 2009; Sasaki et al., 2009; Sunwoo et al., 2009; Zhang et al., 2013; Imamura et al., 2014
EGOT	HCV, Influenza virus, SFV	Up	EGOT	N/A	Favors HCV replication and negatively affects the antiviral response	Carnero et al., 2016
GAS5	HCV	Up	GAS5	Nucleus/Cytoplasm	Inhibited HCV replication by binding viral NS3 protein	Qian et al., 2016
lncRNA#32	EMCV, HBV, HCV	N/A	HECW1	N/A	Interacts with hnRNPU and ATF2 to regulate ISG expression	Nishitsuji et al., 2016
lncBST2	IAV, VSV, HCV	Up	BST2	NA	Controls the potency of the antiviral IFN response	Barriocanal et al., 2015
**VIRUS-ENCODED LncRNAS IN VIRUS-INFECTED CELLS**						
PAN	KSHV	N/A	N/A	Nucleus	A 1.2-kb lncRNA that binds host PABPC1 and is required for the late *KSHV* gene expression. Regulates gene expression through epigenetic mechanisms; interacts with several virus- and host cell-encoded factors; and promotes LANA-episome disassociation through an interaction with LANA	Sun et al., 1996; Ballestas et al., 1999; Borah et al., 2011; Rossetto and Pari, 2011, 2012, 2014; Rossetto et al., 2013, 2016; Campbell et al., 2014; Uppal et al., 2014
β 2.7 RNA	HCMV	N/A	N/A	N/A	Binds directly to GRIM19 to protect virus-infected cells from apoptosis and results in continued ATP production	Greenaway and Wilkinson, 1987; Bergamini et al., 1998; Reeves et al., 2007; White and Spector, 2007; Zhao et al., 2010; Kuan et al., 2012; Poole et al., 2016
sfRNA	Flaviviruses	N/A	N/A	N/A	A 300~500 ntlncRNA generated from incomplete degradation of genomic RNA by the host 5′-3′ exoribonuclease XRN1. sfRNA is involved in viral infection during the innate immune response	Calisher and Gould, 2003; Lin et al., 2004; Knipe and Howley, 2007; Pijlman et al., 2008; Funk et al., 2010; Silva et al., 2010; Chapman et al., 2014; Roby et al., 2014; Clarke et al., 2015; Manokaran et al., 2015; Bavia et al., 2016; Charley and Wilusz, 2016
5.0 kb RNA	HCMV	N/A	N/A	N/A	Highly enriched in AT sequences that lack open reading frames; not required for efficient viral replication in cultured fibroblasts after HCMV infection	Kulesza and Shenk, 2004
7.2 kb RNA	Murine cytomegalovirus	N/A	N/A	Nucleus	Facilitates progression from the acute to the persistent phase of CMV infection	Kulesza and Shenk, 2006; Schwarz et al., 2013; Schwarz and Kulesza, 2014
HIV-expressed antisense lncRNA	HIV	N/A	N/A	N/A	Guides a chromatin-remodeling complex consisting of proteins such as DNMT3a, EZH2, and HDAC-1 to the viral promoter driving transcriptional regulation	Saayman et al., 2014
EBERs	EBV	N/A	N/A	N/A	Play important roles in oncogenesis and antiviral innate immunity *via* theirinteraction with cellular proteins	Kitagawa et al., 2000; Nanbo and Takada, 2002; Nanbo et al., 2002; Samanta et al., 2006, 2008; Iwakiri and Takada, 2010; Iwakiri, 2016
HSURs	Herpes virus	N/A	N/A	N/A	Upregulate the expression of host genes linked to T cell activation in virally transformed T cells,	Lee et al., 1988; Wassarman et al., 1989; Albrecht and Fleckenstein, 1992; Cook et al., 2005; Cazalla and Steitz, 2010; Cazalla et al., 2010
VA RNA	Human adenovirus	N/A	N/A	Cytoplasm	Binds Dicer and functions as a competitive substrate suppressing Dicer to inhibit RNAi. VA RNA also binds, and consequently blocks, PKR activity, inhibits activation of eIF-2a and viral mRNA translation	Mathews and Shenk, 1991; Clarke and Mathews, 1995; Andersson et al., 2005; Xu et al., 2007

*NRAV, negative regulator of antiviral response; NeST, (Nettoie Salmonella pas Theiler's; NRON, non-coding repressor of Nuclear Factor of Activated T cells [NFAT]; NEAT1, nuclear-enriched abundant transcript 1;PAN, Polyadenylated nuclear RNA; sfRNA, subgenomicflavivirus RNA; EBERs, Epstein-Barr virus-encoded RNAs; HSURs, herpes virus saimiri U-rich RNAs; VA RNA, virus-associated RNA I and II encoded by adenovirus; IFN, interferon; SFPQ, splicing factor proline and glutamine rich, a NEAT1-binding paraspeckle protein;PAPBC1, poly (A)-binding protein C1; PKR, protein kinase DAI*.

### Cellular LncRNAs in virus-infected cells

Using Next Generation Sequencing (NGS), differential expression of approximately 500 annotated lncRNAs and 1,000 non-annotated genomic regions after SARS coronavirus infection were identified in mice (Peng et al., 2010). This research represented the first discovery of the widespread differential expression of lncRNAs in response to virus infection and suggested that lncRNAs may be involved in regulating the anti-viral host response. Recently, Qi Zhang et al found 646 lncRNAs were upregulated and 424 lncRNAs were downregulated in latent human cytomegalovirus (CMV) infection on THP-1 cells using RNA-seq analysis (Zhang et al., 2016). However, the critical lncRNA in latent human CMV infection has not been identified and its role has not been elucidated yet. Moreover, additional lncRNAs associated with the virus infection in the host have been identified. Significant differential expression of lncRNAs is induced by virus and regulated by RNA virus or DNA virus infection. In turn, these lncRNAs regulate the host innate immune response including the pathogen recognition receptor (PRR)-related signaling, the production of IFNs and cytokines (Carpenter, 2016; Ouyang et al., 2016). For example, in HCV infection, lncRNA-CMPK2 promotes HCV replication and lncRNA-CMPK2 is significantly upregulated in the primary human hepatocytes after treatment with IFN-α and knockdown of lncRNA-CMPK2 exhibited a negative regulatory role in the modulation of the IFN response with the increase in the expression of several ISGs, such as Mx1, ISG15 and CXCL10 (Kambara et al., 2014). Recently, Carnero et al found HCV infection increased the expression of lncRNA EGOT, an event that was induced by the NF-κB activated retinoic acid-inducible gene 1(RIG-I) and the RNA-activated kinase PKR. Moreover, EGOT expression was also increased after infection with influenza or Semliki Forest virus (SFV) (Carnero et al., 2016). Although, lncRNA EGOT was found to involve in the NF-κB activated RIG-I and PKR pathway, the specific mechanisms in this pathway and antiviral response remain unclear. Additionally, lncRNA GAS5 was found to be upregulated during HCV infection in Huh7 cells and lncRNA GAS5 inhibited HCV replication by binding viral NS3 protein but the innate immune response remains low (Qian et al., 2016).

In influenza virus infection, Ouyang et al found the expression of the lncRNA NRAV was down regulated after infection with a DNA virus (HSV) and with RNA viruses (SeV and MDRV) (Ouyang et al., 2014). NRAV promotes influenza A virus (IAV) replication and virulence and negatively regulates the expression of several critical IFN-stimulated genes (ISGs), including IFIT2, IFIT3, IFITM3, OASL, and MxA. Among these ISGs, the level of MxA was most significantly affected by the expression of NRAV and negatively correlated with NRAV expression. NRAV inhibits the initial transcription of MxA and IFITM3 by regulating histone modifications H3K4me3 and H3K27me3 of the ISG genes. LncBST2/BISPR is expressed from the position in the genome divergent from the well characterized BST2 (a key host cell defense molecule), and lncBST2/BISPR is induced in cells infected with mutants of influenza or VSV. Furthermore, lncBST2/BISPR is upregulated in response to IFN stimulation and was identified as a positive regulator of BST expression. Meanwhile, lncBST2/BISPR is also induced in cells infected with hepatitis C virus (HCV) and in the liver of patients with HCV infections (Barriocanal et al., 2015). Although, lncRNAs, NRAV, and lncBST2, were found in anti-viral response, the specific mechanisms of how they regulate ISG expression have not been elucidated.

LncRNA#32 is 2,946 nt in length and was identified after poly I: C stimulation. The silencing of lncRNA#32 remarkably reduced the level of ISG expression, such as IRF7 and OASL, resulting in sensitivity to encephalomyocarditis virus (EMCV) infection. In contrast, overexpression of lncRNA#32 significantly inhibited EMCV replication. LncRNA#32 interacts with hnRNPU and ATF2 to regulate ISG expression (Nishitsuji et al., 2016). These results suggested that LncRNA#32 was involved in anti-viral responses by controlling ISG expression.

In addition to the roles in the antiviral response, lncRNA NEAT1 is necessary for the formation of the nuclear paraspeckles, unique subnuclear structures for the nucleocytoplasmic transport of mRNA in response to certain stimuli (Clemson et al., 2009; Sasaki et al., 2009; Sunwoo et al., 2009; Naganuma and Hirose, 2013). NEAT1, also known as virus-inducible ncRNA (VINC), was first reported in Japanese encephalitis and rabies virus infections of mice (Saha et al., 2006). The expression of NEAT1 was changed by HIV-1 infection and knockdown of NEAT1 enhanced virus production through increased nuclear to cytoplasmic export of Rev-dependent INS-containing HIV-1 mRNAs (Zhang et al., 2013). In addition, NEAT1 was also induced by influenza virus and HSV infection, and the expression of antiviral genes including cytokines such as IL-8 was facilitated by cooperative action of NEAT1 and SFPQ (splicing factor proline and glutamine rich, a NEAT1-binding paraspeckle protein) (Imamura et al., 2014).

Theiler's picornavirus is a natural pathogen of mice. A mouse lncRNA, NeST (Nettoie Salmonella pas Theiler's, *cleanup* Salmonella not Therler's), was identified in Tmevp3 locus on mouse chromosome10 through gene mapping, which is next to the IFN-γ coding gene *Ifng* (Gomez et al., 2013). The transgenic mouse of T cell specific expression of NeST showed that Theiler's virus increased persistence but decreased *Salmonella enterica* pathogenesis. These observations were likely due to induction of IFN-γ transcription specifically in activated CD8+ T cells by NeST. NeST regulates epigenetic marking of the *Ifng* locus through interaction with a protein partner WDR5, a component of the H3K4 methyltransferase complex. Whether and how disease-associated SNPs alter human NeST expression and/or function has not been elucidated and should be addressed in future studies.

LncRNA NRON (non-coding repressor of NFAT) was initially identified as an inhibitor of transcription factor NFAT (Willingham et al., 2005). NRON interacts with KPNB1, CSE1L, and IQGAP1, which bind phosphorylated NFAT in cytoplasm and represses NFAT nuclear trafficking. When T cells are activated, dephosphorylated NFAT is released from the complex and enters the nucleus (Sharma et al., 2011). This result suggests that lncRNA exists as a scaffold for a latent transcription factor. A recent study suggested that downregulation of NRON by HIV infection enhanced NFAT nuclear translocation and activity (Imam et al., 2015). HIV also utilizes NRON to control the balance between viral reproduction and cell death through the HIV early expressed protein Nef and the late expressed protein Vpu to decrease and increase NRON expression at different infection stages respectively (Imam et al., 2015).

### Virus-encoded lncRNA

During virus infection, the host cell generates various lncRNAs to counteract infection. Similarly, viruses themselves also express many lncRNAs to resist cellular antiviral activity. Here, we describe some virus-encoded lncRNAs that have been identified thus far.

Polyadenylated nuclear RNA (PAN) is encoded by Kaposi sarcoma-associated herpes virus (KSHV) and was first identified as a novel abundant 1.2-kb RNA that is transcribed by RNA Polymerase II (Sun et al., 1996). PAN binds host poly (A)-binding protein C1 (PABPC1) after PABPC1 is translocated to the nucleus during the lytic phase of infection and is required for the late KSHV gene expression, such as vIL-6 and k8.1 (Borah et al., 2011). PAN also interacts with the ORF50 promoter and can either repress gene expression by interacting with protein components of polycomb repression complex 2 (PRC2) to mediate the trimethylation of H3K27 or activate gene expression by interacting with UTX, JMJD3 and the histone methyltransferase MLL2 to mediate the removal of the H3K27me3 mark and simultaneously mark it for activation (Rossetto and Pari, 2012; Rossetto et al., 2013, 2016).

In addition, several virus- and host cell-encoded factors, including histones (H1 and H2A), mitochondrial and cellular single-stranded binding proteins (SSBPs) and interferon regulatory factor 4 (IRF4), interact with PAN (Rossetto and Pari, 2011). LANA is essential for maintaining the episomal form of the viral genome during latency (Ballestas et al., 1999; Uppal et al., 2014). PAN promotes LANA-episome disassociation through an interaction with LANA which facilitates LANA sequestration away from KSHV episomes during reactivation (Campbell et al., 2014). Overall, these studies have revealed that PAN as a major global regulator plays an important role in regulation of viral and host gene expression (Rossetto and Pari, 2014).

Recently, transcriptome analysis lncRNA ALT identified lncRNA ALT as an early lytic transcript and a splice isoform of LANA transcript in KSHV infection (Chandriani et al., 2010; Schifano et al., 2017). The size of lncRNA ALT is large (approximately a ~10,000-nucleotide transcript) and its abundance is very low. However, the specific role of lncRNA ALT remains unclear.

β2.7 RNA, the most abundantly transcribed early gene from the HCMV genome in permissive cells, is a 2.7-kb unspliced polyadenylated lncRNA (Greenaway and Wilkinson, 1987; White and Spector, 2007). Although, it also has some coding potential, β2.7 binds directly to the GRIM19 (genes associated with retinoid/IFN-induced mortality 19), a subunit of mitochondrial enzyme complex I, to protect virus-infected cells from apoptosis and results in continued ATP production, which is critical for the successful completion of the viral life cycle (Bergamini et al., 1998; Reeves et al., 2007). Interaction of the β2.7 RNA with complex I inhibits rotenone stress-induced apoptosis in neuronal cells and this suggests that β2.7 RNA can be exploited in the development of a novel therapeutic for the treatment of Parkinson's disease (Kuan et al., 2012; Poole et al., 2016). Moreover, β2.7 RNA can protect rat aortic endothelial cells from ischemia/reperfusion injury-induced apoptosis by reduction of reactive oxygen species (Zhao et al., 2010).

The subgenomic flavivirus RNA (sfRNA) is 300–500 nt in length and is derived from the 3′ UTR of the RNA genome of flaviviruses, a large group of single-stranded, positive-sense RNA viruses including several human pathogenic viruses, such as yellow fever virus, JEV, dengue viruses, and West Nile virus (Calisher and Gould, 2003; Knipe and Howley, 2007). SfRNA is a product of an incomplete degradation of genomic RNA by the host 5′–3′ exoribonuclease XRN1 and sfRNA is involved in viral infection and host cell response modulation (Roby et al., 2014; Clarke et al., 2015; Bavia et al., 2016; Charley and Wilusz, 2016). The rigid secondary structure stem-loop II located at the beginning of the 3′UTR of the viral genome is resistant to nuclease XRN1 degradation and results in the production of sfRNA (Funk et al., 2010). The sfRNA structure, a ring-like conformation, with the 5′ end of the resistant structure passing through the ring from one side of the fold to the other, is required for the formation sfRNA during flaviviral infection (Chapman et al., 2014). SfRNA generated by the Dengue virus II infection can bind the host proteins G3BP1, G3BP2, and CAPRIN1 and inhibit ISG mRNA translation (Bidet et al., 2014). SfRNA prevents tripartite motif 25 (TRIM25) deubiquitylation, which is critical for sustained and amplified RIG-I-induced type I IFN expression (Manokaran et al., 2015). Production of sfRNA increases the replication efficiency of WNVs and is essential for virus-induced cytotoxicity in cell culture and for viral pathogenicity in mice (Pijlman et al., 2008). However, the mechanisms underlying how sfRNA leads to increased virus replication and cell death remain unknown. SfRNA was also identified in JEV infection and in an RNA pseudoknot that is also necessary for production of yellow fever sfRNA (Lin et al., 2004; Silva et al., 2010).

CMV is a ubiquitous herpes virus that persistently replicates in epithelial cells. A 5-kb immediate-early RNA is a stable intron expressed by human CMV, which is highly AT rich in sequence and lacks open reading frames likely to be translated into protein, and thus is not necessary for efficient replication of the virus in cultured cells after human HCMV infection (Kulesza and Shenk, 2004). A murine CMV 7.2-kb ortholog of the human CMV 5-kb RNA was also identified as a stable intron that facilitates progression from the acute to persistent phase of infection (Kulesza and Shenk, 2006). This CMV lncRNA accumulates in the nucleus of infected cells during infection and whose stability is a result of sustained lariat conformation (Schwarz et al., 2013; Schwarz and Kulesza, 2014).

An HIV-encoded antisense lncRNA without a poly (A) tail was recently discovered. This lncRNA guides a chromatin-remodeling complex consisting of proteins such as DNMT3a, EZH2, and HDAC-1 to the viral promoter driving transcriptional regulation (Saayman et al., 2014).

Although, several ncRNAs are <200 nt in size, Epstein-Barr virus-encoded RNAs (EBERs), herpes virus saimiri U-rich RNAs (HSURs) and virus-associated RNA I and II (VA I and II) encoded by adenovirus are sometimes also referred to as viral lncRNAs.

Two nuclear, highly structured and abundant viral transcripts EBER1 (167 nt) and EBER2 (172 nt) in latently EBV-infected cells are produced by EBV (Iwakiri, 2016). EBER are polyadenylated, ncRNAs that are transcribed by RNA polymerase III (pol III) (Iwakiri, 2016). EBERs play key roles in antiviral innate immunity *via* interaction with cellular proteins (Iwakiri and Takada, 2010). EBERs are recognized by RIG-I and activate its downstream signaling to induce expression of type-I IFNs in EBV-infected cells (Samanta et al., 2006). Furthermore, EBERs induce IL-10 expression through IRF3, but not NF-κB activation, in BL (Burkitt's lymphoma) cells, suggesting that EBER acts as an autocrine growth factor in BL cells(Kitagawa et al., 2000; Samanta et al., 2008). In addition, EBER also contributes to oncogenesis(Nanbo and Takada, 2002). For example, in BL, EBERs counteract IFN-α-induced apoptosis *via* binding to PKR and inhibition of its phosphorylation (Nanbo et al., 2002).

Herpesvirus saimiri, which causes aggressive T-cell leukemia and lymphoma, encodes 7 HSURs (Herpesvirus saimiri (HVS) U-rich RNAs) (Lee et al., 1988; Wassarman et al., 1989; Albrecht and Fleckenstein, 1992). The expression of host genes linked to T cell activation in virally transformed T cells was up regulated by HSURs 1 and 2 (Cook et al., 2005). HSUR 1 directs degradation of host mature miR-27 in a sequence-specific and binding-dependent manner in virally transformed T cells (Cazalla and Steitz, 2010; Cazalla et al., 2010), illustrating a ncRNA to manipulate host-cell gene expression *via* the miRNA pathway after viral infection.

Two highly structured cytoplasmic RNAs; named VA RNA I and VA RNA II (~160–170 nts) are produced from RNA polymerase III (pol III) (Mathews and Shenk, 1991). The VA RNAs bind Dicer and function as competitive substrates suppressing Dicer to inhibit the RNAi (Andersson et al., 2005). Notably, compared with VA RNAI, VA RNA II is incorporated into the RNA-induced silencing complex (RISC) (Xu et al., 2007). Adenovirus VA RNA binds PKR and blocks PKR activity, avoiding phosphorylation of eIF-2a and inhibition of viral mRNA translation (Clarke and Mathews, 1995).

In addition to the cellular lncRNAs and virus encoded lncRNAs, HBx-LINE1 was identified as a chimeric lncRNA, which is produced by viral integration into the host genome leading to activation of a LINE-1 sequence such that a chimeric lncRNA is produced (Lau et al., 2014; Moyo et al., 2016). HBx-LINE functions as an lncRNA-like RNA in HBV-positive HCC cell lines, which induces the Wnt pathway by increasing the nuclear localization of β-catenin. So far, it remains unknown whether other chimeric lncRNAs are identified and their roles in the virus infection.

## Conclusions and perspective

Although, thousands of lncRNAs are expressed after viral infection, the specific lncRNA with experimentally verified functions is limited, thus the roles and functions of lncRNAs in viral infection require further investigation. A deeper understanding of how the lncRNA transcriptome is altered in the infected cell and how these alterations affect the interaction between the host and virus should also be explored. Such studies may help in the identification novel cellular pathways involved in the antivirus response.

In addition to the role of lncRNA associated with the antivirus response, lncRNAs may be both unique diagnostic biomarkers as well as novel targets against which new therapeutics can be developed. Virus-related lncRNAs secreted into the serum may serve as prognostic markers. For example, two serum lncRNAs, uc001ncr and AX800134, have potential as novel potential biomarkers to diagnose HBV-positive HCC, especially in the early stage of disease (Wang et al., 2015). The expression of lncRNA-UCA1 and lncRNA-WRAP53 were significantly higher in sera of HCC than in chronic HCV infection or healthy volunteers (Kamel et al., 2016). This result suggests that lncRNA-UCA1 and lncRNA-WRAP53 upregulation may serve as novel serum biomarkers for HCC diagnosis and prognosis. In conclusion, lncRNAs are key regulators of transcriptional and post-transcriptional processes; thus, their roles in virus infection and therapy necessitate intensive study in the future.

## Author contributions

WL wrote this manuscript and CD designed this project.

### Conflict of interest statement

The authors declare that the research was conducted in the absence of any commercial or financial relationships that could be construed as a potential conflict of interest.
